# *Drosophila* as a Model to Study the Link between Metabolism and Cancer

**DOI:** 10.3390/jdb5040015

**Published:** 2017-12-01

**Authors:** Héctor Herranz, Stephen M. Cohen

**Affiliations:** Department of Cellular and Molecular Medicine, University of Copenhagen, Blegdamsvej 3, 2200 N Copenhagen, Denmark; scohen@sund.ku.dk

**Keywords:** *Drosophila*, animal model, metabolism, cancer, Warburg effect, obesity, type 2 diabetes

## Abstract

Cellular metabolism has recently been recognized as a hallmark of cancer. Investigating the origin and effects of the reprogrammed metabolism of tumor cells, and identifying its genetic mediators, will improve our understanding of how these changes contribute to disease progression and may suggest new approaches to therapy. *Drosophila melanogaster* is emerging as a valuable model to study multiple aspects of tumor formation and malignant transformation. In this review, we discuss the use of *Drosophila* as model to study how changes in cellular metabolism, as well as metabolic disease, contribute to cancer.

## 1. Introduction

Cancer is a genetic disease that involves the accumulation of mutations that affect multiple biological processes. Cancer-promoting changes include sustaining proliferative signalling, evading growth suppressors, resisting cell death, enabling replicative immortality, inducing angiogenesis, and activating invasion and metastasis, which can result in the death of the host [1]. Tumorigenesis is also dependent on changes in cellular metabolism. Nearly a century ago, Otto Warburg observed a metabolic shift in cancer cells toward increased use of glucose in a process called aerobic glycolysis. However, tumor metabolism has only recently been recognized as a hallmark of cancer [1,2]. Interest in cancer metabolism has grown rapidly in recent years, with an emphasis on understanding how cancer metabolism is regulated, how it influences disease progression, and how these factors could be exploited to improve cancer therapy [3].

*Drosophila melanogaster* is emerging as a valuable model for studying neurological disorders, cardiovascular diseases, and cancer. Flies have many experimental advantages, including sophisticated genetic tools, a short life cycle, and the ease with which large numbers of individuals can be examined in well-targeted genetic screens. Genetic conservation between flies and humans is substantial, with 75% of known human disease genes having related sequences in *Drosophila* [4]. Consequently, hypotheses generated using flies often prove to be relevant to biomedicine. *Drosophila* has served as a platform to develop models that recapitulate various aspects of human cancer, and its use has contributed to revealing different aspects of human malignant tumors [5]. This review summarizes the contribution of *Drosophila* to study how metabolism affects tumorigenesis and malignant transformation.

## 2. Metabolic Control of Tissue Growth

Nutritional status plays a pivotal role regulating metabolism and tissue growth during development. Nutrient-sensing systems coordinate growth by connecting nutrient availability and metabolism with the growth machinery. Insulin/insulin-like growth factor (IGF) signalling is a universal, nutrient-sensing system. It is conserved from mammals to *Drosophila* and plays a central role regulating growth in response to nutrient availability. insulin/IGF binds receptor tyrosine kinases, and this leads to the activation of the Ras/MAPK and PI-3K/Akt/TOR pathways. By activating those signalling pathways, insulin/IGF coordinates multiple processes frequently deregulated in cancer, including cellular growth, proliferation, metabolism, and cell survival. Key elements of these signalling cascades, including PI-3K, Akt, TOR, and PTEN, are recognized oncogenes and tumor suppressors [6]. Research in *Drosophila* has served to determine how components of the insulin/IGF system, including Rheb, Tsc1, and Tsc2, are organized in the insulin/Akt pathway, and has contributed to defining how insulin/Akt controls systemic and cellular growth. Those studies (reviewed in depth elsewhere [7,8,9]) have expanded our knowledge of the normal physiological roles that these oncogenes and tumor suppressors play, as well as how their dysregulation promotes disease.

## 3. Warburg Effect in *Drosophila*

Nearly a century ago Otto Warburg observed that tumor cells had altered glucose metabolism. In the presence of oxygen, normal cells convert glucose to pyruvate through glycolysis. Pyruvate dehydrogenase (PDH) mediates the conversion of Pyruvate to Acetyl-CoA, which feeds into the Tricarboxylic acid (TCA) cycle and will eventually be metabolized to CO_2_ in the mitochondria via oxidative phosphorylation. Under anaerobic conditions, pyruvate is converted to lactate by lactate dehydrogenase (LDH). Warburg observed that tumor cells use glycolysis rather than the TCA cycle and oxidative phosphorylation, even in the presence of oxygen [10,11]. This, ultimately leads to the generation of lactate by the tumor cells and is known as aerobic glycolysis, and the ability of cancer cells to obtain this metabolic state was termed Warburg effect. Recent studies have noted that the Warburg effect contributes to cancer progression [2].

LDH is a key enzyme in this process. Human LDH enzymes are encoded by four separate genes, LDHA-D, which catalyse the conversion of pyruvate to lactate. The LDHA isoform is predominantly expressed in cancer cells and has higher affinity for pyruvate, favouring the conversion of pyruvate to lactate [12,13]. Tumor cells frequently grow in an oxygen-poor environment and hypoxia has been proposed as the primary signal that induces metabolic reprogramming in tumor cells [14]. In an oxygen-poor environment, the Hypoxia inducible factor-1α (Hif-1α in *Drosophila*) is stabilized. At normal oxygen levels, Hif-1α is rapidly degraded. Hif-1α has been shown to regulate LDHA expression in cancer cell lines [15]. Even though this supports the metabolic switch observed in hypoxia, it does not explain how aerobic glycolysis is maintained in normoxic conditions. LDH is encoded by a single gene (*ImpL3*) in *Drosophila*. Biochemical studies demonstrated that fly LDH, like mammalian LDHA, favors the conversion of pyruvate to lactate [16]. The presence of a single LDH gene in flies eliminates potential genetic redundancy of the LDH family members and facilitates the analysis of its function. This, together with the sophisticated toolkit available in *Drosophila*, has allowed the use of *Drosophila* to study the Warburg effect.

### 3.1. Warburg Effect in Drosophila Tumors

The *Drosophila* imaginal discs are epithelial structures that proliferate extensively during larval development and, after metamorphosis, give rise to the external structures of the adult fly. The imaginal discs have been used to model different aspects of tumorigenesis [17]. Activation of the PDGF/VEGF-receptor (Pvr) in the imaginal discs leads to the formation of epithelial tumors that up-regulate LDH and show aerobic glycolysis [18,19]. Pvr activation in the fly imaginal epithelium has therefore been used as a model to dissect how aerobic glycolysis is induced and maintained in epithelial tumor cells [19]. Pvr activation causes metabolic reprogramming by affecting glycolysis and mitochondrial respiration. On the one hand, cells activating Pvr upregulate LDH and several glycolytic enzymes in a Hif-1α dependent manner. On the other hand, Pvr attenuates mitochondrial respiration by downregulating genes encoding mitochondria proteins, and by inhibiting PDH activity. One of the primary consequences of mitochondrial dysfunction is the accumulation of reactive oxygen species (ROS) [20,21]. ROS accumulation has two important outcomes: (1) it stabilizes Hif-1α; and (2) ROS activates the c-Jun amino (N)-terminal kinase pathway (JNK) pathway, which has been shown to inhibit PDH activity [22,23]. Therefore, experiments performed in *Drosophila* have demonstrated that the accumulation of ROS in tumor cells is used as a feedback mechanism to consolidate the high-LDH/low-PDH state, and thereby to stabilize the aerobic glycolysis state.

The Notch signalling pathway regulates cell proliferation and differentiation, and has been shown to function as both an oncogene or as a tumor suppressor [24,25]. Notch behaves as an oncogene in the *Drosophila* imaginal discs, in which Notch activation leads to tissue hyperplasia and tumor formation [26]. Notch activation also induces a switch towards increased glycolysis and reduced TCA cycle and mitochondrial respiration in the imaginal epithelia. Interestingly, several key metabolic regulators like the glucose transporter Glut-1, the glycolytic enzyme hexokinase A, and LDH have been shown to be direct target genes of the Notch pathway [27].

These studies have shed light on the molecular mechanisms by which the Pvr and Notch oncogenes promote a shift toward Warburg effect metabolism in epithelial tumors [19,27].

### 3.2. Warburg Effect in Normal Development

Warburg effect metabolism is thought to promote cancer cell growth by diverting glucose metabolism toward production of amino acids at the expense of efficiency in ATP production via the TCA cycle [28]. Although apparently inefficient, this shift favors increased biomass by the converting nutrients into cellular building blocks to support rapid growth during normal larval development in the fly. This depends on the activity of the *Drosophila* estrogen-related receptor (dERR) [29], a nuclear receptor that regulates the expression of genes involved in glucose metabolism. dERR mutants show lower expression of almost all glycolytic genes and die in the larval stage, presenting high levels of circulating glucose and reduced levels of lactate and triacylglycerides. The metabolic state induced by dERR aims to support larval growth by generating biomass from dietary carbohydrates, and it resembles the metabolic switch observed in Warburg effect. Interestingly, multiple studies have implicated human ERRs in cancer [30,31,32,33]. The results obtained in *Drosophila* raise the possibility that ERRs might control tumor development, at least in part, by modulating cellular metabolism in a manner that promotes a Warburg-like phenotype.

Recent work has used the *Drosophila* intestinal stem cell model to analyse how changes in the glucose metabolism affect intestinal homeostasis. The *Drosophila* gut is a simple system in which intestinal stem cells divide to self-renew and to give rise to a class of progenitors called enteroblasts, which differentiate (without cell division) into two different cell lineages: absorptive intestinal epithelial cells (enterocytes) and endocrine cells [34,35,36]. The mitochondrial pyruvate carrier (MPC) mediates pyruvate uptake by the mitochondria and is crucially linked to glycolysis and mitochondrial oxidation. In the *Drosophila* intestine, MPC shows high levels of expression in the more differentiated endocrine cells, and its expression is lower in the proliferative stem cells. Interestingly, MPC mutant intestinal cells proliferate faster than their wild type counterparts, and guts mutant for MPC develop tumorous growths with epithelial multi-layering, characteristics of cancer. Reducing LDH levels in the pool of intestinal stem cells has the opposite effect and leads to a reduction in the proliferation rate [37]. These results demonstrate that pyruvate oxidative metabolism plays a central role in regulating stem cell proliferation, differentiation, and tissue homeostasis, as well as in providing additional evidence supporting a central role of metabolism in development and tumorigenesis.

### 3.3. Mitochondrial Dysfunction and Tumor Progression

Pyruvate produced in glycolysis is used in the mitochondria to generate ATP and metabolites. Mitochondrial dysfunction is common in human cancers. However, whether mitochondrial dysfunction is a consequence of the increase in the glycolytic flux, or whether it is a driving force in tumorigenesis, is an ongoing subject of debate [38,39,40,41]. The Ras^V12^ oncogenic mutation induces cell proliferation and benign tumors in *Drosophila* epithelial cells [42] and has been used as a sensitized genetic background to search for cooperating factors that promote formation of neoplastic tumors [43,44,45]. One screen identified multiple mitochondrial genes that, when mutated, cooperate with Ras^V12^ in the formation of malignant tumors and metastasis [44]. Interestingly, while the genetically modified cells induce p53 and undergo cell cycle arrest and Ras-induced cellular senescence [46], they act in a non-autonomous manner to drive the formation of tumors in the surrounding normal cells [44]. These surprising findings may make sense in light of recent studies showing that senescent cells secrete cytokines and growth factors known as the senescence-associated secretory phenotype (SASP) [47]. Senescence, therefore, has concurrent and opposing roles: it has an autonomous suppressive role aimed at eliminating tumor cells, and a non-autonomous signalling effect mediated by the SASP phenotype that can promote overproliferation, leading to tumor formation in nearby cells.

*Drosophila* genetics has been used to explore the molecular connection between mitochondrial dysfunction and the SASP phenotype. *Drosophila* epithelial cells defective in mitochondrial function and expressing Ras^V12^ produce ROS, which activates the JNK stress-response pathway. This represses the Hippo tumor-suppressor pathway and leads to upregulation of the secreted proteins Unpaired/interleukin-6 and Wingless/WNT. Unpaired and Wingless induce cell proliferation and benign hyperplasia of neighbouring tissue. Interestingly, when Ras^V12^ is active in the surrounding cells, activation of the Unpaired and Wingless pathways promotes malignant tumors and metastasis [44]. These observations suggest that mitochondrial defects, rather than being a consequence of the increase in the glycolytic flux observed in cancer cells, can play an instructive role in cancer progression. These studies may provide a context to understand the effects of mutations in respiratory-chain complex genes that have been observed in pancreatic cancer [40], which, in most cases, also have activating mutations in Ras [48].

## 4. The Link between Metabolic Diseases and Cancer

In addition to changes in cellular metabolism, metabolic dysfunctions can influence cancer. Obesity is a complex metabolic disorder that results in excessive accumulation of dietary lipids. Changes in lifestyle, linked to increased consumption of energy rich foods and more sedentary behaviour, increase the incidence of obesity-associated metabolic disease, including type 2 diabetes. There is a strong clinical association between these metabolic disorders and the risk of specific types of cancer, and patients with metabolic dysfunction have higher cancer-related mortality [49,50,51,52,53]. However, the mechanisms that link metabolic morbidities and cancer are not well understood. We discuss here how *Drosophila* has been used to investigate the obesity-cancer interaction.

### 4.1. Factors Controlling Obesity

In addition to the impact of changes in life style, human obesity has a strong genetic component [54]. Genome-wide screens using *Drosophila* have been used to identify novel genetic factors that can contribute to obesity [55]. An RNAi-based in vivo screen identified ~500 genes that led to changes in fat levels in adult flies. 60% of these genes have human orthologues. Depletion of those candidates, specifically in the fly adipose tissue, created a role for the conserved hedgehog (hh) signalling pathway. Downregulation of the hedgehog pathway in the fat body, via the depletion of *hh*, *iHog*, and *smoothened*, or the transcription pathway *cubitus interruptus*, led to a marked increase in triglyceride levels. In a mouse model, fat-specific hedgehog-activation led to lean mice with a strong reduction in fat tissue, revealing conservation between flies and mammals in adipocyte tissue physiology [55].

Genome-wide transcriptome profiling comparing starved versus fed flies implicated the lipase gene *brummer* (*bmm*) in obesity [56]. The expression of *bmm* is controlled by the nutritional status of the animal. *bmm* downregulation is sufficient to cause obesity and, reciprocally, flies overexpressing *bmm* showed a depletion in organismal fat stores, resembling nutrient-deprived animals. *bmm* encodes the lipid-storage, droplet-associated triacylglycerol lipase, which is a homolog of the human adipocyte triglyceride lipase. The obesity-related phenotype observed in flies depleted of *bmm*, together with this evolutionary conservation, suggest that regulation of this family of genes could be involved in human obesity [56].

In addition to genetics and the environment, studies in human populations have demonstrated that the parental nutritional state can influence the metabolism of the offspring for multiple generations [57,58,59,60]. Even though different molecular mechanisms for imprinting, including DNA methylation, histone modifications, and non-coding RNA have been implicated in this process [61], how intergenerational phenotypes are initiated, stabilized, and transmitted remains largely unknown. As in humans, the nutritional state of the parents affects the metabolism of the offspring in flies: an increase in paternal dietary sugar affects metabolism in the offspring. The analysis of flies grown in higher sugar diet (HSD) detected changes in the chromatin state of mature sperm and in the embryos they produce that led to obesity in the offspring. Interestingly, many genes involved in cytosolic and mitochondrial metabolism are found in those chromatin regions, which might account for the metabolic changes observed in the offspring. The use of a *Drosophila* has allowed the identification of gene networks involved in parental-induced intergenerational metabolic reprogramming. This gene signature is conserved from flies to mice and humans [62], suggesting that similar gene networks may regulate obesity susceptibility in humans and *Drosophila*.

### 4.2. Connecting Obesity and Cancer

There is a clinical association between obesity and several types of cancer. A primary consequence of obesity is the development of insulin-resistant type 2 diabetes. When the body becomes resistant to insulin, it compensates by producing more insulin [63]. In addition to its glucose regulatory function, insulin is a mitogen that promotes tissue growth [64], and, thus, chronic elevation of systemic insulin levels is a candidate for promoting tumorigenesis in obese and diabetic patients.

The insulin pathway is conserved from flies to humans, and feeding flies with HSD induces hyperinsulinemia and insulin resistance [65], which resembles the metabolic defects observed in obese humans. Activation of the oncogenes Ras and Src is common in multiple human cancers [66], and this genetic background has been used in *Drosophila* to study epithelial tumor formation [67]. Clones of cells activating Ras and Src in the imaginal discs of *Drosophila* do not develop into tumors when raised on a control-normal diet; however, they form malignant tumors with aggressive metastatic-like behaviour when the flies are fed on a HSD [68]. While most tissues in flies fed in HSD are insulin-resistant, cells activating the oncogenes Ras-Src express high levels of the insulin receptor. This makes these tumors sensitive to the elevated levels of systemic insulin produced by these nutritional conditions. High insulin signalling in the Ras-Src-expressing cells upregulates the signalling molecule Wingless (WNT), which, in turn, causes a further increase in Insulin receptor levels, creating a positive feedback loop that reinforces this program. The increase of insulin receptor levels in Ras-Src tumors makes them sensitive to insulin, allowing them to use circulating sugar to fuel their growth [68].

How do tumors in animals fed on high sugar trigger the initial upregulation of the insulin receptors to benefit from high systemic insulin? Further studies by the Cagan group identified the salt-inducible kinases (SIKs) as proteins induced by activation of Ras and Src in flies fed in HSD [69]. SIKs are serine/threonine kinases of the AMP kinase family of proteins that control metabolic homeostasis in mammals and flies [70,71,72,73,74,75]. SIKs can induce tissue growth by repressing the Hippo tumor suppressor pathway [76]. HSD induces SIKs in cells activating the oncogenes Ras and Src. This inhibits the Hippo kinase cascade that results in the activation of the Hippo transcriptional activator, the oncogene Yorkie, which induces the expression of its target gene Wingless [69]. SIKs act as sugar sensors that link the nutritional status to Hippo activity. By using this mechanism, the Ras-Src cells respond to changes in the diet to promote tumor growth in conditions of HSD. The finding that diet influences tumor development in *Drosophila* opens the possibility to use genetic tools to further analyse the molecular links between obesity and the risk of cancer.

### 4.3. Drosophila Fat Body to Identify Drugs that Modulate Lipid Metabolism

*Drosophila* has also been used as a platform to search for chemical compounds regulating signalling pathways involved in cancer and metabolism [77]. WNT signalling, in addition to its implications in specific types of cancer, has been shown to control adipocyte development [78,79]. Adipocytes play central roles in controlling fat metabolism and storage in mammals. The adipocytes in the *Drosophila* fat body contain numerous lipid droplets and store most of the triglycerides of the fly, having a similar metabolic role to mammalian adipose tissues. Axin is a negative regulator of the Wingless cascade in *Drosophila*, and *Axin* depletion results in an increase in Wingless pathway activity. Interestingly, *Axin* mutants show reduced triglycerides and a smaller amount of fat body tissue, and the adipocytes in *Axin* mutants are reduced in size and contain fewer oil droplets. The *Axin* mutant background was used to screen for compounds that regulate the Wingless pathway and lipid metabolism. That screening resulted in the identification of peptide boronic acids (a family of proteasome inhibitors) as chemical compounds that reduce Wingless signalling and rescue the fat body defects observed in *Axin* mutants [77]. This work exemplifies how fly models can be used to identify candidate drugs.

### 4.4. TGF-βs as Regulators of Obesity and Insulin Resistance

A high-fat diet can also induce type 2 diabetes in humans. Adipose tissue, in addition to functioning as the site of fat storage, behaves as an endocrine organ that secretes signalling molecules that control metabolic homeostasis [80,81]. The signalling molecule TGF-β is secreted by the adipose tissue and has been identified as a critical mediator of diet-induced insulin resistance in mice, although the mechanism by which TGF-β controlled insulin resistance was not elucidated [82]. In *Drosophila*, a high fat diet (HFD) leads to elevated circulating triglyceride and glucose levels, and insulin resistance, akin to obese mammals. This has allowed the use of *Drosophila* genetics to explore diet-induced insulin resistance [83]. The *Drosophila* gene *glass bottom boat* (*gbb*) is a TGF-β family member with functions in growth, morphogenesis, and energy homeostasis [84,85,86,87]. Consistent with the result obtained in mice, the protein levels of the TGF-β Gbb are increased in HFD conditions, and overexpression of *gbb* in the *Drosophila* fat body is sufficient to trigger obesity and insulin resistance, recapitulating the HFD phenotypes. Conversely, depletion of *gbb* results in a rescue of the HFD-induced metabolic phenotypes. Gbb signalling promotes insulin resistance by increasing the expression of the gene *tribbles* [88]. TRB3, a mammalian homolog of *Drosophila tribbles*, functions as a negative modulator of Insulin pathway in mice by regulating the serine-threonine kinase Akt, which is a central mediator of insulin signalling. TRB3 physically interacts with Akt and represses its kinase activity, causing insulin resistance [89]. In agreement with those observations, *Drosophila* Tribbles represses Akt in the fat body. HFD-fed mice upregulate TGF-β and the orthologous protein TRB3, which inhibited insulin signalling. The combination of studies in mice and flies shed light on the mechanisms by which TGF-β controls obesity and diabetic phenotypes [88,89]. 

Defects in mitochondrial activity have been associated with obesity, and both mitochondrial perturbations and obesity influence cancer progression. Accumulating evidence shows that mitochondrial alterations in one organ can have systemic effects through hormonal signalling [90,91]. *Drosophila* has been used to explore how mitochondrial defects can modulate fat accumulation in distant organs. This analysis found that mitochondrial perturbations in the muscle can influence mitochondrial function and metabolism in the fat body, leading to triglyceride accumulation, characteristic of obesity [83]. This work identified the TGF-β ligand, Activin, as the key element mediating mitochondrial synchrony between the muscle and the fat body. *Activin* is expressed at low levels in wild-type muscles, but its expression is increased in muscles with defects in mitochondrial activity. Activin secreted by the muscles acts systemically to impair mitochondrial activity and increase lipid accumulation in the fat body. This work identified a molecular mechanism whereby Activin behaved as a hormone to regulate “mitochondrial synchrony” between organs. The results obtained in *Drosophila* are in good agreement with previous observations showing that Activin represses mitochondrial function and lipid accumulation in mammals [92], and they suggest that this mechanism is conserved.

## 5. *Drosophila* as a Model to Study Cachexia

More than 80% of advanced human cancer patients show signs of loss of body mass caused by degeneration of skeletal muscle and fat storage wasting, a disorder known as cachexia [93]. Cachexia remains as a major hurdle in cancer treatment, in part because the molecular mechanisms that drive wasting remain elusive. As in humans, the presence of malignant tumors in *Drosophila* impairs muscle function and causes systemic wasting [94,95]. The induction of neoplastic tumors in flies leads to metabolic reprograming in the muscle that results in a strong downregulation of genes involved in energy metabolism, including glycolysis, TCA cycle, and Oxidative phosphorylation. This leads to mitochondrial degeneration and a reduction in ATP levels in muscle cells. *Drosophila* tumors produce *ImpL2*, a homolog of the secreted insulin growth factor-binding protein. Elevated ImpL2 release can reduce systemic insulin signalling in flies with malignant tumors. Depletion of insulin activity is sufficient to induce cachexia-like phenotype. Notably, suppression of ImpL2 increases insulin signalling and alleviates some wasting phenotypes associated with these tumors [94,95], suggesting a potential therapeutic approach to cachexia.

## 6. Metabolism and Angiogenesis

Angiogenesis is the physiological process through which new blood vasculature is formed. Tumors often grow in nutrient- and oxygen-poor environments, and angiogenesis is one of the strategies used to obtain nutrients and oxygen. Consequently, angiogenesis is a key target in cancer treatment [1]. In *Drosophila*, the tracheal system has an analogous role to the mammalian circulatory system, providing internal organs with oxygen [96,97]. Similar to vertebrate capillaries, the tracheal system responds to low oxygen levels by generating new branches via Hif-1α and Fibroblast Growth Factor signalling [98,99]. Angiogenesis also operates in neoplastic *Drosophila* tumors [100]. Angiogenesis is thought to be driven by signals secreted by oxygen-deprived target cells. However, a recent study in *Drosophila* has identified a new mechanism by which nutritional status regulates angiogenesis in the *Drosophila* larval gut [101]. Tracheal tubes in the gut are in close proximity to axons that emanate from insulin-producing neurons in the *Drosophila* brain. Those neurons guide changes in tracheal architecture through expression of Insulin-like peptide 7 (Ilp7), as well as pigment dispersing factor (Pdf), a neuropeptide that shares similarities to the vertebrate Vasoactive Intestinal Polypeptide. Ilp7 and Pdf control the growth of the tracheal cell terminal branches in response to nutritional cues received by those neurons. This work illustrates a new mechanism that couples the metabolic state with the process of angiogenesis. It will be of interest to see whether future studies show similar links between systemic metabolism and angiogenesis in human cancer progression.

## 7. Metabolism and Cell Competition

Tumors have several strategies, beyond angiogenesis, for obtaining nutrients. Cancer cells can use amino acids produced from the degradation of extracellular proteins internalized via macropicnocytosis, from entosis of living cells, from phagocytosis of apoptotic bodies, and from lipids from the extracellular phospholipids [2]. For example, cells activating the oncogene K-Ras kill and engulf surrounding cells by a process known as cell competition [102], and accumulating evidence suggests that cell competition contributes to cancer [103,104,105,106]. Cell competition was first described in *Drosophila* in the 1970s [107] and serves as a fitness selection mechanism by which “more fit” cells (winners) recognize and eliminate their “less fit” neighbours (losers) [105].

Metabolic pathways active in tumor cells are controlled by oncogenes [108,109], and oncogene activation can trigger cell competition [110]. Oncogenic Ras promotes cell growth and proliferation in *Drosophila* by activating the PI-3K pathway and by increasing Myc protein levels [111]. *Drosophila* cells expressing high levels of the *Myc* oncogene acquire a “supercompetitor” status and induce apoptosis in normal surrounding cells that, when surrounded by those supercompetitor cells, become losers [112,113]. Myc controls cellular metabolism in different ways. In the *Drosophila* fat tissue, Myc regulates glucose and lipid metabolism to favor the storage of nutrients lipids and sugars [114]. In a context of cell competition, *Myc*-expressing cells reprogram their metabolism towards enhancing glycolysis in a p53-dependent manner. In absence of p53, cells upregulating Myc lose their supercompetitor status and cannot induce apoptosis in surrounding normal cells, so that the population of *Myc*-expressing cells cannot increase in size [115]. This suggests that metabolic reprogramming might play a crucial role in early phases of disease, whereby emerging cancer cells might expand by boosting glycolysis.

Cell competition involves the induction of apoptosis in the loser cells and, in some cases, this is accompanied by the engulfment of the losers by the winners [102,104,116,117]. A recent study identified the conserved miRNA, *miR-8*, as a cooperative partner of the *Epidermal Growth Factor Receptor* (*EGFR*) oncogene [104]. Coexpression of *EGFR* and *miR-8* in the imaginal wing epithelium results in the formation of metastatic tumors. An interesting characteristic of those tumors is that they contain giant polyploid cells, which induce apoptosis and engulf their neighbouring cells. Interestingly, inhibition of apoptosis or engulfment completely suppress the formation of the polyploid cells and, consequently, tumors and metastasis are not formed. These observations suggest that cancer polyploid cells may need to engulf surrounding cells in order to obtain nutrients that allow them to grow and become malignant [104].

Downregulation of the gene *calderón* (*cald*) also induces cell competition in *Drosophila* epithelia. *cald* encodes a protein with homology on the major facilitator super-family of organic cation transporters, and these transporters are believed to function mainly in the uptake of sugars and other organic metabolites. The *cald* gene is regulated by the insulin pathway and is required for insulin-regulated cell growth and proliferation. Interestingly, cells mutant for *cald* are viable when surrounded by other *cald* mutant cells, but they are eliminated by cell competition when their neighbours are normal cells [118]. This report suggests that the metabolic state of the cell can impact in the “winner” vs. “loser” status and hence induce cell competition and tumor progression. 

## 8. Perspectives

In this review, we have discussed how the use of *Drosophila melanogaster* has contributed to the study of mechanistic links between metabolism and cancer. Metabolic changes are emerging as important drivers of malignancy, and cancer-specific metabolic alterations are becoming an important research theme. *Drosophila* has been broadly used to study how metabolism is orchestrated to support biological processes, including growth, proliferation, and apoptosis in normal development. The fact that *Drosophila* shares a great degree of gene conservation with humans has made of this model organism a valuable tool for the identification and analysis of genes involved in cancer metabolism. To date, most studies have been focused on alterations in glucose metabolism. However, cancer cells also use nutrients like glutamine, amino acids, essential fatty acids, etc. Given the underlying similarity in metabolism, future studies using fly models should be used to explore links between nutrient utilization and disease progression. A variety of obesity-related models have been developed in *Drosophila*. The combination of these with available fly tumor models should be a fruitful path to investigate the molecular connections between diet, obesity, diabetes, and cancer. Finally, the impact of metabolites produced by gut microbiota on tumor progression is only starting to be appreciated. *Drosophila* has been extensively used to investigate the contribution of microbiota on insect behaviour, immunity, and metabolism. This will allow the fly to be used to study the influence that metabolic changes mediated by the activity of the microbiota have on disease progression.
